# “Childhood overweight and obesity: maternal perceptions of the time for engaging in child weight management”

**DOI:** 10.1186/1471-2458-12-295

**Published:** 2012-04-20

**Authors:** Petra Warschburger, Katja Kröller

**Affiliations:** 1 Department of Psychology, University of Potsdam, Karl-Liebknecht-Str. 24/25, 14476, Potsdam, Germany

**Keywords:** Maternal perception, Need for action, Prevention, Obesity, Overweight, Children

## Abstract

**Background:**

There is an increasing awareness of the impact of parental risk perception on the weight course of the child and the parent’s readiness to engage in preventive efforts, but only less is known about factors related to the parental perception of the right time for the implementation of preventive activities. The aim of this study was to examine parental perceptions of the appropriate time to engage in child weight management strategies, and the factors associated with different weight points at which mothers recognize the need for preventive actions.

**Methods:**

352 mothers with children aged 2–10 years took part in the study. We assessed mothers’ perceptions of the actual and preferred weight status of their child, their ability to identify overweight and knowledge of its associated health risks, as well as perceptions of the right time for action to prevent overweight in their child. A regression analysis was conducted to examine whether demographic and weight related factors as well as the maternal general risk perception were associated with recognizing the need to implement prevention strategies.

**Results:**

Although most of the parents considered a BMI in the 75^th^ to 90^th^ percentile a valid reason to engage in the prevention of overweight, 19% of the mothers were not willing to engage in prevention until their child reached the 97^th^ percentile. Whereas the child’s sex and the identification of an elevated BMI were significant predictors for parents’ recognition of the 75^th^ percentile as right point to engage in prevention efforts, an inability to recognize physical health risks associated with overweight silhouettes emerged as a significant factor predicting which parents would delay prevention efforts until a child’s BMI reached the 97^th^ percentile.

**Conclusion:**

Parental misperceptions of overweight and associated health risks constitute unfavorable conditions for preventive actions. Feedback on the health risks associated with overweight could help increase maternal readiness for change.

## Background

The prevention of overweight is a high-priority public health task. According to a recent epidemiological survey in Germany (2003 – 2006), 3% of 3- to 6-year-old children and even 6.4% of 7- to 10-year-olds qualify as obese, while 6% and 9% of these groups, respectively, are overweight [1]. Childhood obesity is associated not only with an increased risk of several physical morbidities, but can also lead to serious psychological problems [2]. Moreover, unusual weight gain in early infancy has been identified as the main risk factor for the development of later overweight [3,4].

Parents control many aspects of their child’s environment, and are therefore vital to the implementation of recommended prevention strategies. The majority of research on this topic has been conducted to explore how parents can support their child’s weight loss or maintenance of a healthy weight. However, parents frequently do not perceive the need for preventive actions. According to psychological health theories, the perception of risks is an essential requirement for health behavior change. As numerous studies have shown, many parents often fail to recognize the overweight or at-risk status of their child and the associated health problems [5-8]. Factors predicting the underestimation of weight and risk status of the child are the child’s young age, rapid weight gain in infancy, the mother’s and child’s higher weight status, as well as lower educational attainment and an African-American background [9-15]. The correct identification of a child’s weight status seems to be relevant if any change in weight is recommended: in a prospective study, Kroke et al. [16] showed that children who were correctly identified as overweight at 6 months of age by their mothers had a greater decrease in the standardized BMI (BMI-SDS) than those who were not correctly identified.

Accurate weight estimation seems to be necessary but not sufficient for employing prevention or intervention strategies. Myers and Vargas [17] found that 37% of parents, though correctly recognizing their child’s overweight, had nevertheless taken no action to change it. One explanation for this discrepancy is parental failure to associate their child’s weight status with higher health risks. We are only aware of two studies exploring factors associated with parental readiness for change. Rhee, DeLago, Arscott-Mills, Mehta and Davis [18] focused on parents with overweight children aged 2 to 12 years: 44% were not interested in making lifestyle changes to help their child lose weight within the next 6 months. Aside from the child’s age, higher readiness for change was associated with the belief that their child’s weight constituted a health problem. Crawford, Timperio, Telford and Salford [19] even reported that parental concern about the future – but not the actual weight status of their child – increased parents’ use of prevention strategies.

The discrepancy between the parental preferred and perceived weight status of their child is an additional motivational incentive discussed in the literature. A parental preference for their child to have a slender silhouette has been associated with an earlier perception of the need for preventive activities [20-22].

There is an increasing awareness of the impact of parental risk perception on the weight course of the child and the parent’s readiness to engage in preventive efforts. Although a few studies have pointed out that risk perception does not necessarily lead to intervention, less is known about factors related to the parental perception of the right time for the implementation of preventive activities. The objectives of the present study were to determine at which weight status mothers perceived that actions for preventing their child’s overweight became necessary, as well as the factors associated with different weight statuses at which mothers perceive the need for prevention efforts. Based on the existing research we hypothesized that parents would decide that action was needed based on their general perception of the risk associated with overweight, their own weight status and that of the child, as well as maternal dissatisfaction with the child’s current weight status.

## Methods

### Procedure

As part of a larger study of childhood obesity prevention (supported by BMBF^a^ project number 01EL0408), a questionnaire was administered to mothers between February and October 2007. Participants were recruited in roughly equal parts from inpatient clinics (e.g. those specializing in child rehabilitation for respiratory diseases, dermatitis or susceptibility to infections), child care centers, and online communities. Eligible participants were mothers of children aged one to ten who had no health-related dietary restrictions. In order to make participation more convenient, the questionnaire was available either online or in a pencil-and-paper version, and participants were free to choose their preferred format. Depending on the place from which the mothers were recruited, they filled in the paper-and-pencil version in the clinic or child care center and returned it to the office, they filled it out at home and returned it later, or they completed the online version, which could be filled in and sent from their personal computer. On average, mothers needed fifteen minutes to finish the questionnaire, which included about 120 items. Mothers who have more children in the indicated age range were requested to select one, that we could analyze mother-child pairs.

This study was approved by the University of Potsdam institutional ethics committee and has therefore been performed in accordance with the ethical standards of the 1964 Declaration of Helsinki.

### Measures

#### Socio-demographic variables

Mothers provided details concerning their marital status, age and education as well as their child’s sex and age. Family’s net income (including unemployment, housing, maternity or sick leave benefits, pensions, and wages or comparable earnings) was calculated, taking into account the current number of family members in the household. The graduation level is the number of years of school the mother completed.

#### Weight status

All mothers reported their own height and weight as well as the height and weight of their child. Accordingly, these reported heights and weights were used to calculate subjects’ BMIs. For children, we used BMI percentiles and a standardized BMI (BMI-SDS) according to age and sex [23]. The classification of children as overweight (BMI percentile > 90) was done according to national guidelines [24].

#### Perception of weight status and need for action

To assess the perception of weight status, mothers were presented with a panel of silhouettes (described in more detail in [12]). These were based on relevant anthropometric measures of children in different age groups (2- to 4-years or 5- to 10-years old) and of different genders (female or male). In each set, two silhouettes represented underweight children (3^rd^ and 10^th^ percentile), three sketches represented normal weight children (25^th^, 50^th^ and 75^th^ percentile), and two represented an overweight or obese child (90^th^ and 97^th^ percentile). A previous study validates these silhouettes as a tool for the assessment of weight perceptions across the represented age and gender groups [25].

Mothers were asked to answer the following questions in relation to the age- and gender-relevant set of silhouettes for their own child: “Which of the silhouettes do you think represent overweight children?” (identification of overweight silhouettes), “Which silhouette best represents the weight status of your child?” (perception of their own child) and “Which of the silhouettes do you prefer for your child?” (preferred body size). The difference between the current and preferred weight status was operationalized as a measure of weight dissatisfaction. Additionally, mothers were questioned about their perception of risks of physical and mental health problems associated with overweight (e.g. “Which silhouettes do you think bear an increased risk for physical/mental health problems?”), as well as their perception of the need for action to prevent obesity (“Which of the silhouettes signals your readiness to employ strategies to prevent overweight in your child?”). Mothers were able to answer all of these questions by indicating multiple silhouettes, except for the perceived current weight status and the preferred body size of their child. In the analyses we report, we consider the first mark set as a lower bound.

### Sample

A total of 482 mother-child pairs fulfilled the inclusion criteria (age range of 2 to 10 years, with no diet-related health problems). 130 participants were excluded because their questionnaires were more than 5% incomplete. There were no significant differences in demographic and weight variables between non-completers and completers. The final sample for statistical analysis consisted of 352 participants (151 (43%) mothers who filled in the online questionnaire, and 201 (57%) mothers who filled in the paper version, in which 32% were recruited from child-care centers and 68% from children’s health clinics). 95.4% of the mothers were German citizens and 78.3% lived in a partnership. Their children averaged 5.67 years of age (range from 2 to 10 years), and their distribution of sexes was almost equal. Table 1 summarizes the demographic aspects and the weights of the participants. Preliminary analyses revealed differences between mothers who filled in the online questionnaire and those who were recruited from clinics or child-care centers: mothers who were recruited from child-care centers had younger children on average than those who filled in the online version or were recruited from children’s health clinics (*F*(1, 349) = 24.68, *p* < .01). Additionally, mothers who filled in the online questionnaire reported lower weights for their child (*F*(1, 349) = 8.91, *p* < .01) and for themselves (*F*(1, 349) = 12.54, *p* < .01) as well as a higher level of education (*F*(1, 349) = 22,89, *p* < .01) than mothers who were recruited from clinics. These variables were included as covariates in the regression reported below. Differences regarding the recruitment strategies were controlled accordingly.

**Table 1:** Sample description

**Children (n = 352)**	
**Sex**	168 (47%) female; 184 (53%) male
**Age**	M = 5.67 years, SD = 1.97 (2 – 10 years of age) - 40 (12%) under 3 years of age - 159 (45%) between 3 and 6 years of age - 153 (33%) over 6 years of age
**BMI-SDS** (mother’s report)	M = −0.26, SD = 1.30 (− 5.41 – 4.07) - 57 (16%) underweight (BMI-SDS < 10^th^ perc.) - 261 (74%) normal weight (10^th^ perc. ≥ BMI-SDS ≤ 90^th^ perc.) - 34 (10%) overweight or obese (BMI-SDS > 90^th^ perc.)
**Mothers (n = 352)**	
**Age**	M = 34.48 years, SD = 5.20 (20 – 50 years of age)
**Per capita income** (per month)	M = 739.57 Euro, SD = 295.53 (220 – 2667 Euro)
**educational level** (years in school)	M = 10.89 school years, SD = 1.40 (6 – 12 years of school)
**BM I** (self report)	BMI = 24.37 kg/m^2^, SD = 5.60 (15.89 – 50.77 kg/m^2^) - 20 (6%) underweight (BMI < 18.5 kg/m^2^) - 214 (61%) normal weight (18.5 kg/m^2^ ≥ BMI ≤ 25 kg/m^2^) - 118 (33%) overweight or obese (BMI > 25 kg/m^2^)

### Statistical analyses

All analyses were performed using SPSS 20.0 (Chicago: SPSS inc.). Because missing data rates were below 5% and missing analyses did not show any associations between missing values and included variables, Expectation Maximization substitution was applied for metric scales. Based on the marked silhouettes, the identification of overweight and the associated health risk, as well as the perceived need for preventive action were grouped as follows: at the 75^th^ percentile or before, at the 90^th^ percentile and at the 97^th^ percentile. We submitted the data on demographic and weight differences regarding the recruitment strategies as well as the maternal ability to identify overweight silhouettes and their perception of the need for preventive activities to ANOVAs and *χ*^2^ tests where appropriate. To analyze factors associated with maternal desire to engage in child weight management at different weight points, we used a multinomial logistic regression analysis. Using the 90^th^ percentile as a reference point, we contrast an early-action need (operationalized as the 75^th^ BMI percentile or lower) and an already-too-late action need (operationalized as the 97^th^ BMI percentile) as represented by the silhouettes. Based on previous research, demographic and weight aspects (child’s sex and age, weight status of mother and child, maternal education and income level), maternal dissatisfaction with the child’s silhouettes (satisfied/preferred heavier silhouettes/preferred thinner silhouettes) as well as their ability to identify overweight silhouettes (75^th^ percentile/90^th^ percentile/97^th^ percentile) and the related health risks (75^th^ percentile or before/90^th^ or 97^th^ percentile) were included in the prediction model.

## Results

### Identification of overweight silhouettes and related health risks

Overall, 150 mothers (43%) identified the overweight silhouettes (90^th^ and 97^th^ percentile) correctly, and 56 mothers (16%) identified only the 97^th^ percentile as overweight (see Table 2). Demographic and weight related influences produced no significant differences in the maternal ability to identify overweight silhouettes (*χ*^2^ (df = 1) 0.01 – 3.25; p = .92 - .07). Only 50% of mothers believed that silhouettes above the 90^th^ percentile carry a higher physical risk, and only 46% believed that these silhouettes were related with higher mental health risk. Mothers who were able to identify these health risks correctly were more often higher educated (*χ*_physical_^2^ (df = 1) 4.40, *p* = .04; *χ*_mental_^2^ (df = 1) 5.00, *p* = .03) or had overweight children (*χ*_physical_^2^ (df = 1) 4.54, *p* = .03; *χ*_mental_^2^ (df = 1) 3.75, *p* = .05).

**Table 2:** Identification of overweight silhouettes, the related health risk and perceived need for action

	**Percentile:**			
	**50.**	**75.**	**90.**	**97.**
Identification of overweight	n = 14 (4%)	n = 137 (38%)	n = 153 (42%)	n = 56 (16%)
Identification of mental health risk	0	n = 31 (14%)	n = 111 (50%)	n = 80 (36%)
Identification of physical health risk	0	n = 43 (20%)	n = 111 (50%)	n = 65 (30%)
Perceived need for action	n = 16 (4%)	n = 92 (26%)	n = 179 (51%)	n = 65 (19%)

### Dissatisfaction with the child’s silhouette

Regarding the discrepancy between the ideal and the actual body size, most parents were content with the current figure of their child (62%). Of those who were not, the majority preferred a slightly heavier (24%) than a thinner (14%) silhouette for their child. This maternal dissatisfaction with the child’s body size differs depending on the child’s age and weight as well as the mother’s weight. Mothers of older children more often preferred a thinner silhouette for their child than mothers of younger children (*χ*^2^ (df = 2) 11.64; p < .01). Additionally, mothers who had an overweight child (*χ*^2^ (df = 2) 137.16; p < .01) or were overweight themselves (*χ*^2^ (df = 2) 23.07; p < .01) preferred thinner silhouettes.

### The perception of the need for action to prevent overweight

In general, mothers pointed out different silhouettes as the right indications of prevention efforts. 51% of the mothers indicated the silhouette of the 90^th^ percentile as a starting point for engaging in child weight management. 19% indicated no need for action until the 97^th^ percentile, and 30% indicated silhouettes in the 75^th^ or 50^th^ percentile as needing professional help (see Table 2). The maternal perception of the need for preventive actions differs only with respect to child’s age: mothers of younger children indicated the need for weight management strategies for heavier silhouettes than mothers of older children (*χ*^2^ (df = 2) 18.95; p < .01).

### Prediction model

To predict the maternal recognition of the need for preventive actions in silhouettes of children in the 75^th^ to 97^th^ percentiles, we used a multinomial logistic regression including demographic variables (sex, weight, educational level and income), maternal perception of overweight, maternal perception of the associated health risks, and weight dissatisfaction (see Table 3). This model shows an acceptable fit to our data (*R*^*2*^, Nagelkerke’s method = .43). Apart from the child’s gender, an earlier maternal perception of need for action was more likely if mothers identified overweight at or before the 75^th^ percentile. So, the probability for an earlier perception of need for preventive actions was three times less for mothers with female children, but 13 times higher for mothers who identified overweight at the 75^th^ percentile. A later maternal perception of the need for preventive activities was less likely if mothers were aware of the associated physical health risks in overweight silhouettes – that is that the likelihood for a later perception of need for preventive actions is four times less for mothers who identified with overweight associated physical health risks at the 90^th^ and 97^th^ percentile. All other variables were found to be non-significant.

**Table 3:** Predicting the maternal perception for the need of action at the 75^th^ and at the 97^th^ percentile

**Need for action on silhouettes on the 75**^**th**^**percentile:**	**B**	**SD** **error**	**Wald**	**df**	**p**	**OR**	**CI**
**Child’s sex:***female*	**−1.05**	**.51**	**4.23**	1	**.04**	**0.35**	**0.13 – 0.95**
**Child’s age**	0.09	.14	0.46	1	.50	1.10	0.84 – 1.44
**Child’s weight**	−0.18	.26	0.49	1	.48	0.84	0.51 – 1.39
**Mother’s weight**	−0.02	.05	0.13	1	.72	0.98	0.90 – 1.08
**Mother’s education**	−0.07	.20	0.14	1	.71	0.93	0.63 – 1.38
**Family income**	0.00	.00	0.03	1	.87	1.00	1.00 – 0.95
**Weight dissatisfaction** *- satisfaction* *- preferred heavier silhouette*	−0.30 −1.31	.88 1.02	0.12 1.64	1 1	.73 .20	0.74 0.27	0.13 – 4.15 0.04 – 2.00
**Identification of overweight silhouettes:***- 75*^*th*^* percentile* * - 90*^*th*^* percentile*	**2.57** 0.63	**.92** .97	**7.79** 0.42	1 1	**.01** .52	**13.12** 1.87	**2.15 – 79.96** 0.28 – 12.56
**Identification of physical health risk:***90*^*th*^* and 97 *^*th*^* percentile*	−0.95	.50	3.61	1	.06	0.39	0.15 – 1.03
**Identification of mental health risk:***90*^*th*^* and 97*^*th*^* percentile*	−0.57	.49	1.36	1	.24	0.57	0.22 – 1.47
**Need for action on silhouettes on the 97**^**th**^**percentile:**	**B**	**SD ** **error**	**Wald**	**df**	**p**	**OR**	**CI**
**Child’s sex:***female*	0.53	.53	0.99	1	.32	1.70	0.60 – 4.81
**Child’s age**	−0.19	.14	1.73	1	.19	0.83	0.63 – 1.10
**Child’s weight**	−0.25	.24	1.11	1	.29	0.78	0.49 – 1.24
**Mother’s weight**	−0.05	.05	1.23	1	.27	0.95	0.87 – 1.04
**Mother’s education**	−0.21	.20	1.09	1	.30	0.82	0.56 – 1.20
**Family income**	0.00	.00	0.05	1	.82	1.00	1.00 – 1.00
**Weight dissatisfaction** *- satisfaction* *- preferred heavier silhouette*	−0.81 −1.15	.91 1.07	0.78 1.15	1 1	.38 .28	0.45 0.32	0.08 – 2.67 0.04 – 2.59
**Identification of overweight silhouettes:***- 75*^*th*^* percentile* * - 90*^*th*^* percentile*	−0.86 −0.48	.75 .68	1.34 0.51	1 1	.25 .48	0.42 0.62	0.09 – 1.82 0.16 – 2.34
**Identification of physical health risk:***90*^*th*^* and 97*^*th*^* percentile*	**−1.38**	**.57**	**6.00**	**1**	**.01**	**0.25**	**0.08 – 0.76**
**Identification of mental health risk:***90*^*th*^* and 97*^*th*^* percentile*	−0.69	.56	1.51	1	.22	0.50	0.17 – 1.51

## Discussion

On one hand, health professionals urge that prevention of childhood overweight should begin early and focus on parents as the primary agents of change. On the other hand we know almost nothing about what weight parents consider to be a “warning sign” that they should engage in preventive weight management efforts with their children. Based on past research on parental risk perception, this study first explored parental perceptions of the right time for engaging in activities to prevent their child from being overweight, and second, analyzed which factors predict parents’ sensitivity to the degree of their child’s risk – e.g. whether they see a concrete need for action at a lower weight status, or only at a higher weight status. Within this context we further investigated parental risk perception as well as their dissatisfaction with their child’s weight.

Half of mothers correctly identified the overweight silhouettes as well as related health risks, but 16 - 20% of mothers only identified overweight and its associated health risks with silhouettes corresponding to BMIs in the 97^th^ percentile. These results are in agreement with other studies’ reports of low parental risk perceptions of children’s weight status [e.g. 6, 11]. The associated physical and mental health problems were correctly classified more often by mothers with higher educational attainment and already-overweight children. Maternal education as a factor influencing the ability to recognize overweight-related health problems was previously shown in our own [12] and other studies [summarized in 11]. The child’s overweight status has so far only been examined as a factor increasing maternal tendencies to misclassify overweight in her own child [9,10], but there were no associations in this study between the child’s weight and maternal misclassification of unrelated silhouettes [12]. Therefore, for mothers of overweight children it may be easier to apply knowledge about the obesity-related health risks to unrelated children rather than their own. Further studies that focus on this question are needed.

Concerning maternal dissatisfaction with her own child’s silhouette, our results are in line with the work of Laraway and colleagues [21]. More mothers preferred a slightly thicker than a thinner silhouette for their child, and the desire for a thinner silhouette increased with the child’s overweight status. This desire seems to make sense, since we did not explicitly focus on already-overweight children, and therefore most children were in the normal weight range. Mother’s own overweight status and the age of the child were associated with a maternal preference for a thinner silhouette. In our opinion these results can be interpreted as possible outcome of more experience with obesity in both cases. Even though there is a higher prevalence of overweight in older children [1], the child’s weight status is not the underlying factor in this relation between the child’s age and maternal preference for thinner silhouettes. However, overweight mothers could be more aware of having an overweight child due to their own experiences with overweight. This view is supported by the observation that overweight mothers reported more concern about their child’s future weight status independent of the child’s current weight status [26].

Half of the mothers see the need for action only on overweight silhouettes (90^th^ percentile). In addition, 20% of mothers are convinced that there is no need for prevention until the child’s BMI exceeds the 97^th^ percentile, i.e. when he or she is already obese. Taken together, these results emphasize the fact that the vast majority of parents do not share basic ideas of health promotion or primary prevention with health professionals, whose standards state that prevention should start early and before the child suffers from a weight problem. This observation is consistent with the low participation rates often reported in prevention studies [27]. The only significant factor to predict this late parental engagement in preventive efforts (at the 97^th^ percentile) was maternal inability to identify physical health risks with overweight and obese silhouettes. This finding underscores the approach of common health-related psychological theories (e.g. the Health Action Process Approach of Schwarzer or the Theory of Reasoned Action of Ajzen; see [28]) which stress the relevance of risk perception as a necessary basis for the construction of an intention to change behavior. Current empirical research reveals that parental identification of overweight, as well as parents’ beliefs that overweight constitutes a health problem, are highly significant influences [18,29] in prevention and intervention. Since most mothers underestimate the health risks associated with overweight, there is an urgent need to sensitize parents to the health risks related to childhood obesity.

Maternal commitment to preventive activities at the 75^th^ weight percentile, on the other hand, is predicted by having a boy and by the identification of overweight at the 75^th^ percentile. Previous work has already reported the importance of early parental identification of overweight, as well as the identification of related health risks, as relevant factors affecting parental readiness for prevention activities with respect to maternal risk perception [summarizing 11]. Our finding that mothers see an earlier need for action for boys is unexpected. Since we controlled for several factors, this result could not be explained simply by the higher weight status of the boys, or by age differences. There are only a few studies that find significant sex differences regarding parental risk perception and these show the opposite results with respect to gender (in summary [11]). In addition, we did not examine maternal perceptions of preventive activities for their own child, but in general for children in this age group. Therefore, we would not have expected any associations with the gender of the participant’s own child. We are not aware of any study using a similar approach to bring our results in line with other research. Further investigations are needed to explore the influence of the child’s gender on maternal risk perception.

Our study has several limitations. We used a cross-sectional design, which does not allow us to draw causal conclusions. Further prospective research is therefore required to confirm the effects of the child’s age, as well as maternal ability to recognize overweight and related health risks in unrelated children. Another limitation is that we used self-report data for weight status. This decision was supported by our assumption that maternal perception of the child’s weight status is more relevant to their perception of weight-related health risks than his or her objective weight.

Still, our study is the first to focus on the maternal perception of the need for overweight prevention. Our results underscore the relevance of parental risk perception to the construction of intentions to employ preventive strategies.

## Conclusions

This study suggests that most parents believe there is no need for preventive action regarding their child’s weight until the child is overweight or even obese. The early identification of overweight in children and its related physical health risks are factors that can increase parental motivation for preventive actions. In summary, these findings underscore the relevance of parental awareness about overweight. Preventive efforts should take this into account and should develop methods to raise parental risk awareness as a first step towards decreasing children’s health risks from overweight and obesity. First results show that parents prefer a clear statement about their child’s weight status [30], but especially for younger children only few parents being told by their doctor that their child is overweight [31,32]. Additionally, getting feedback about the child’s weight status by pediatricians or other health care professionals or recalling a doctor’s concern and advice will increase their awareness on overweight associated health risks [33-35] and also their willingness to change family habits [36,37]. Therefore further research is necessary how to support pediatricians in screening at risk children and in counseling their parents (summarizing [38]). Otherwise it seems unlikely that their will implement any relevant intervention targeting overweight.

## Endnotes

^a^This is the German acronym for the Federal Ministry of Education and Research in Germany.

## Competing interests

Both authors declare that they have no competing interests.

## Authors’ contribution

PW conceived of the study, participated in its design and coordination, steered the statistical analysis and drafted the manuscript. KK participated in the design of the study and coordination, performed the statistical analysis and drafted the manuscript. Both authors read and approved the final manuscript.

## Pre-publication history

The pre-publication history for this paper can be accessed here:

http://www.biomedcentral.com/1471-2458/12/295/prepub

## References

[B1] Kurth B-M Schaffrath Rosario A Die Verbreitung von Übergewicht und Adipositas bei Kindern und Jugendlichen in Deutschland Bundesgesundheitsbl-Gesundheitsforsch-Gesundheitsschutz 2007 5 736 743 10.1007/s00103-007-0235-5 17514458

[B2] Reilly JJ Methven E McDowell ZC Hacking B Alexander D Stewart L Kelnar CJH Health consequences of obesity Arch Dis Child 2003 88 748 752 10.1136/adc.88.9.748 12937090 PMC1719633

[B3] Parsons TJ Power C Logan S Summerbell CD Childhood predictors of adult obesity: a systematic review Int J Obes Relat Metab Disord 1999 23 8 1 107 10641588

[B4] Whitaker RC Wright JA Pepe MS Seidel KD Dietz WH Predicting obesity in young adulthood from childhood and parental obesity N Engl J Med 1997 337 13 869 873 10.1056/NEJM199709253371301 9302300

[B5] Cottrell LA Minor V Murphy E Ward A Elliott E Tillis G Turner M Neal WA Comparisons of parent cardiovascular knowledge, attitudes, and behaviors based on screening and perceived child risks J Community Health Nurs 2007 24 87 99 10.1080/07370010701316213 17563281

[B6] Etelson D Brand DA Patrick PA Shirali A Childhood Obesity: Do parents recognize this health risk? Obes Res 2003 11 1362 1368 10.1038/oby.2003.184 14627757

[B7] Chaparro MP Langellier BA Kim LP Whaley SE Predictors of Accurate Maternal Perception of Their Preschool Child's Weight Status Among Hispanic WIC Participants Obesity (Silver Spring) 2011 10.1038/oby.2011.105 21546937

[B8] Oude Luttikhuis HG Stolk RP Sauer PJ How do parents of 4- to 5-year-old children perceive the weight of their children? Acta Paediatr 2010 99 2 263 267 19900176 10.1111/j.1651-2227.2009.01576.x

[B9] Jackson J Strauss CC Lee AA Hunter K Parents' accuracy in estimating child weight status Addict Behav 1990 15 65 68 10.1016/0306-4603(90)90007-K 2316412

[B10] Mamun AE McDermott BM O’Callaghan MJ Najman JM Williams GM Predictors of maternal misclassification of their offspring’s weight status: a longitudinal study Int J Obes 2008 32 48 54 10.1038/sj.ijo.0803757 18193064

[B11] Towns N D’Auria J Parental perceptions of their child's overweight: An integrative review of the literature J Pediatr Nurs 2009 24 2 115 130 10.1016/j.pedn.2008.02.032 19268233

[B12] Warschburger P Kröller K Maternal perception of weight status and health risks associated with obesity in children Pediatrics 2009 124 1 e60 e68 10.1542/peds.2008-1845 19564270

[B13] Baughcum AE Chamberlin LA Deeks CM Powers SW Whitaker RC Maternal perceptions of overweight preschool children Pediatrics 2000 106 1380 1386 10.1542/peds.106.6.1380 11099592

[B14] Manios Y Moschonis G Grammatikaki E Anastasiadou A Liarigkovinos T Determinants of childhood obesity and association with maternal perceptions of their children's weight status: the "GENESIS" study J Am Diet Assoc 2010 110 10 1527 1531 10.1016/j.jada.2010.07.004 20869492

[B15] West DS Raczynski JM Phillips MM Bursac Z Gauss CH Montgomery BEE Parental recognition of overweight in school-age children Obesity 2008 16 630 636 10.1038/oby.2007.108 18239596

[B16] Kroke A Strathmann S Gunther ALD Maternal perceptions of her child’s body weight in infancy and early childhood and their relation to body weight status at age 7 Eur J Pediatr 2006 165 12 875 883 10.1007/s00431-006-0191-3 16896647

[B17] Myers S Vargas Z Parental perceptions of the preschool obese child Pediatr Nurs 2000 26 1 23 30 12026313

[B18] Rhee KE DeLago CW Arscott-Mills T Mehta SD Davis RK Factors associated with parental readiness to make changes for overweight children Pediatrics 2005 116 1 e94 101 10.1542/peds.2004-2479 15995022

[B19] Crawford D Timperio A Telford A Salford J Parental concerns about childhood obesity and the strategies employed to prevent unhealthy weight gain in children Public Health Nutr 2006 9 7 889 895 17010255 10.1017/phn2005917

[B20] Contento IR Basch C Zybert P Body image, weight, and food choices of Latina women and their young children J Nutr Educ Behav 2003 35 5 236 248 10.1016/S1499-4046(06)60054-7 14521823

[B21] Laraway KA Birch LL Shaffer ML Paul IM Parent perception of healthy infant and toddler growth Clin Pediatr 2009 49 4 343 349 10.1177/0009922809343717 PMC3623679 19745095

[B22] Zalilah MS Anida HA Merlin A Parental perceptions of children's body shapes Med.J.Malaysi 2003 58 5 743 751 15190662

[B23] Kromeyer-Hauschild K Wabitsch M Kunze D Geller F Geiß HC Hesse V von Hippel A Jaeger U Johnsen D Korte W Menner K Müller G Müller JM Niemann-Pilatus A Remer T Schaefer F Wittchen H-U Zabransky S Zellner K Ziegler A Hebebrand J Perzentile für den Body-Mass-Index für das Kindes- und Jugendalter unter Heranziehung verschiedener deutscher Stichproben Monatsschrift Kinderheilkd 2001 149 807 818 10.1007/s001120170107

[B24] Zellner K Jaeger U Kromeyer-Hausschild K Height, weight and BMI of schoolchildren in Jena, Germany – are the secular changes levelling off? Econ Hum Biol 2004 2 281 294 10.1016/j.ehb.2004.04.006 15464007

[B25] Warschburger P prep Die Entwicklung und Validierung von Körpersilhouetten für Vorschulkinder

[B26] Kämpfe S Parental perspectives of child weight status and potential health risks. Unpublished thesis 2010

[B27] Young-Hyman D Herman LJ Scott DL Schlundt DG Care giver perception of children´s obesity-related health risk: A study of African American families Obes Res 2000 8 241 248 10.1038/oby.2000.28 10832767

[B28] Conner M, Norman P Predicting health behaviors 2 Open University Press, Maidenhead, England 223 275

[B29] Wee CC Davis RB Phillips RS Stage of readiness to control weight and adopt weight control behaviors in primary care J Gen Intern Med 2005 20 5 410 415 10.1111/j.1525-1497.2005.0074.x 15963162 PMC1490121

[B30] Bolling C Crosby L Boles R Stark L How pediatricians can improve diet and activitity for overweight preschoolers: a qualitative study of parental attitudes Acad Pediatr 2009 9 3 172 178 10.1016/j.acap.2009.01.010 19450777 PMC4374621

[B31] Barlow SE Dietz WH Management of Child and Adolescent Obesity: Summary and Recommendations Based on reports From Pediatricians, Pediatric Nurse Practitioners, and registered Dietitians Pediatr 2002 110 236 238 12094001

[B32] Perrin EM Skinner AC Steiner MJ Parental recall of Doctor communication of weight status Arch Pediatr Adolesc Med 2011 10.1001/archpediatrics.2011.1135 PMC3368219 22147758

[B33] Carnell S Edwards C Croker H Boniface D Wardle J Parental perceptions of overweight in 3–5 y olds Int J Obes 2005 2 353 355 10.1038/sj.ijo.0802889 15768040

[B34] Hernandez RG Cheng TL Serwint JR Parents‘healthy weight perceptions and preferences regarding obesity counseling in preschoolers: Pediatricians matter Clin Pediatr 49 790 798 10.1177/0009922810368288 20522610

[B35] Perrin EM Jacobson JC Benjamin JT Skinner AC Wegner S Ammerman AS Use of Pediatrician toolkit to address parental perception of children’s weight status, nutrition, and activity behaviors Acad Pediatr 10 4 274 281 20554259 10.1016/j.acap.2010.03.006 PMC2897945

[B36] Barlow SE Bobra SR Elliott MB Brownson RC Haire-Joshu D Recognition of childhood overweight during health supervision visits: Does BMI help pediatricians? Obes 15 1 225 232 10.1038/oby.2007.535 17228051

[B37] Schwartz RP Hamre R Dietz WH Wasserman RC Slora EJ Myers EF Sullivan S Rockett H Thoma KA Dumitru G Resnicow KA Office-based motivational interviewing to prevent childhood obesity: a feasibility study Arch Pediatr Adolesc Med 2007 161 5 495 501 10.1001/archpedi.161.5.495 17485627

[B38] Perrin EM Finkle JP Benjamin JT Obesity prevention and the primary care pediatrician’s office Curr Opin Pediatr 19 3 354 361 17505200 10.1097/MOP.0b013e328151c3e9 PMC2692353

